# Human cardiomyocytes are more susceptible to irreversible electroporation by pulsed electric field than human esophageal cells

**DOI:** 10.14814/phy2.15493

**Published:** 2022-10-27

**Authors:** Maura Casciola, Devin Keck, Tromondae K. Feaster, Ksenia Blinova

**Affiliations:** ^1^ Division of Biomedical Physics, Office of Science and Engineering Laboratories, Center for Devices and Radiological Health US Food and Drug Administration Silver Spring Maryland USA

**Keywords:** atrial fibrillation, human esophageal smooth muscle cells (hESMCs), human‐induced pluripotent stem cell‐derived cardiomyocytes (hiPSC‐CMs), human in vitro assay, irreversible electroporation (IRE), pulsed field ablation (PFA)

## Abstract

Pulse electric field‐based (PEF) ablation is a technique whereby short high‐intensity electric fields inducing irreversible electroporation (IRE) are applied to various tissues. Here, we implemented a standardized in vitro model to compare the effects of biphasic symmetrical pulses (100 pulses, 1–10 μs phase duration (d), 10–1000 Hz pulse repetition rate (f)) using two different human cellular models: human‐induced pluripotent stem cell‐derived cardiomyocytes (hiPSC‐CMs) and human esophageal smooth muscle cells (hESMCs) cultured in monolayer format. We report the PEF‐induced irreversibly electroporated cell monolayer areas and the corresponding electric field thresholds (EFTs) for both cardiac and esophageal cultures. Our results suggest marked cell type specificity with EFT estimated to be 2–2.5 times lower in hiPSC‐CMs than in hESMCs when subjected to identical PEF treatments (e.g., 0.90 vs 1.85 kV/cm for the treatment of 100 pulses with d = 5 μs, f = 10 Hz, and 0.65 vs 1.67 kV/cm for the treatment of 100 pulses with d = 10 μs, f = 10 Hz). PEF treatment can result in increased temperature around the stimulating electrodes and lead to unanticipated thermal tissue damage that is proportional to the peak temperature rise and to the duration of the PEF‐induced elevated temperatures. In our study, temperature increases ranged from less than 1°C to as high as 30°C, however, all temperature changes were transient and quickly returned to baseline and the highest observed ∆T returned to 50% of its maximum recorded temperature in tens of seconds.

## Graphical abstract

Esophageal fistula is a rare, but life‐threatening risk associated with traditional thermal cardiac ablation procedure in atrial fibrillation patients. Irreversible electroporation (IRE) is a new approach to induce tissue ablation used by novel pulsed electric field (PEF) devices that has demonstrated some tissue specificity in animal models. Here, for the first time, we demonstrate that the electroporation thresholds are lower for human cardiomyocytes than for human esophageal cells for a range of PEF parameters using a well‐controlled in vitro assay. This result may support and improve understanding or the potential superior safety of the IRE‐based cardiac ablation devices.

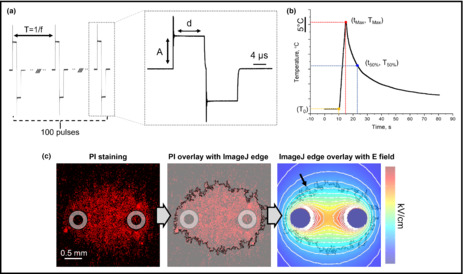

## 1 INTRODUCTION

Thermal catheter ablation techniques, including radiofrequency (RF) ablation and cryoablation, are the current gold standard for the treatment of medication‐resistant atrial fibrillation (Calvert et al., 2022; Habibi et al., 2021). This technique relies on the application of extreme heat or cold to damage the aberrant heart tissue and block the abnormal arrhythmia‐inducing conductance. The long‐term success rate in arrhythmia treatment is reported to be 50%–64% for patients receiving a single RF ablation treatment, while a higher success rate of 65%–77% is reported for patients receiving multiple RF ablation treatments (Calkins et al., 2009). RF ablation attempts to localize thermal energy in the arrhythmic substrate; however, thermal energy lacks tissue specificity and on occasion can lead to collateral tissue damage when temperature gradients travel beyond the desired ablation site. Major periprocedural complications were reported in 5% of patients treated using RF ablation (Calkins et al., 2009). These rare but potentially fatal complications including off‐target tissue damage like left atrium‐esophageal fistula (Kapur et al., 2017) and phrenic nerve damage (Sacher et al., 2007) could be potentially minimized if cardiac ablation was intrinsically limited to heart tissue.

Tissue specificity is one potential advantage of the novel approaches to cardiac ablation based on cell electroporation (Cochet et al., 2021; Howard et al., 2020; Koruth et al., 2020; Nakatani et al., 2021; Stewart et al., 2021). Nonthermal, irreversible electroporation (IRE) leading to cardiac cell death can be induced by the application of short, high‐intensity pulsed electric fields (PEF) to a target area (Maor et al., 2019; Reddy et al., 2019; Reddy et al., 2020; Stewart et al., 2019; Sugrue et al., 2022; Verma et al., 2021). A PEF treatment is characterized by several parameters, including pulse shape (e.g., rectangular uni‐phasic or biphasic), phase amplitude (A), phase duration (d), interphase interval, pulse repetition rate (f), number of pulses delivered in one train (N), number of trains, etc. Depending on the parameters selected, PEF can induce a cell response that results in reversible (acute) or irreversible (long‐term) effects (Davalos et al., 2005; Gudvangen et al., 2022; Zupanic et al., 2012). Thus, ablation treatment by IRE is enabled through the application of PEF that, if properly selected, results in irreversible cell damage leading to death by apoptosis and necrosis (Batista Napotnik et al., 2021; Davalos et al., 2005).

Although IRE ablation devices are in advanced clinical developmental stages, the evidence for the tissue specificity of the PEF‐induced electroporation is limited. Many studies have been performed to characterize the IRE electric field thresholds (EFT) in different animal models, for various tissue types, including myocardium, pancreas, kidney, liver, vascular smooth muscles, and nerves (Arena et al., 2012; Avazzadeh et al., 2021; Kaminska et al., 2012; Li et al., 2011; Maor et al., 2009; Neal et al., 2015; Neven et al., 2017; Sano et al., 2010). Unfortunately, a direct comparison of the IRE EFT values from these sources is usually not appropriate due to the differences in experimental protocols and various animal species used. A more systematic approach was taken to compare the IRE effects on the pancreas, liver, and brain using a combination of pig tissue experiments and computer modeling (Beitel‐White et al., 2021). A recent study focused on cardiac ablation, compared cell death in neonatal rat ventricular cardiomyocytes, rat cortical neurons, and esophageal smooth muscle cells exposed to the same PEF treatment (Hunter et al., 2021). While this study showed a higher sensitivity of cardiomyocytes compared with neurons and esophageal cells, it was limited to one PEF treatment (i.e., symmetrical square biphasic pulses with a fixed phase duration of 5 ms at increasing pulse amplitudes).

Human cell lines of cancerous and noncancerous origin were used to assess selective sensitivity to PEF treatments (Aycock et al., 2022; Baena‐Montes et al., 2022; Ĉemazˆr et al., 1998; Gianulis et al., 2017). However, to the best of our knowledge, no attempt has been made to compare IRE EFT in human cardiac and esophageal cells, in part due to the lack of an appropriate in vitro model prior to the discovery of human‐induced pluripotent stem cell‐derived cardiomyocytes (hiPSC‐CMs). Over the last decade, hiPSC‐CMs use in medical product development has increased and they are now widely available from multiple commercial sources. Moreover, they have been thoroughly validated for studying the effects of drugs on cellular electrophysiology (Blinova et al., 2017; Blinova et al., 2018; Strauss et al., 2019) and are being applied to the safety assessment of cardiac electrophysiology medical devices (Blinova et al., 2019; Casciola et al., 2020; Feaster et al., 2021).

Here, we report a standardized in vitro model based on human cells to compare, for the first time, IRE effects for two different spatially adjacent tissues repressing the heart and esophagus under comparable experimental conditions. We used 4‐h posttreatment Propidium Iodide (PI) uptake as a sensitive indicator of cell death. We imaged PEF‐induced regions of irreversible cell electroporation stained with PI, quantified their areas (i.e., IRE area), and evaluated the IRE EFTs in human cardiac and esophageal cells for a range of treatment parameters (100 pulses, d = 1–10 μs, f = 10–1000 Hz) as well as measured thermal changes for these pulsing conditions.

## 2 MATERIALS AND METHODS

### 2.1 Cell culture and maintenance

Cryopreserved human esophageal smooth muscle cells (hESMC) (catalog #2710, ScienCell Research Laboratories, Carlsbad, CA) were plated according to the manufacturer's protocol onto a 2 μg/cm^2^ poly‐L‐lysine‐coated vented culture flask and maintained at 37°C, with 5% CO_2_. Complete Smooth Muscle Cell Medium (catalog # 1101, ScienCell Research Laboratories) was changed every 3 days. Cells were passaged when the culture reached 90–95% confluency. For experiments, hESMCs were dissociated and removed from the vented culture flask and plated on a 2 μg/cm^2^ poly‐L‐lysine coated 96‐well Nanofiber plates (catalog # 9602, Nanofiber Solutions, Dublin, OH) at a concentration of 100,000 cells per well to reach 100% cell confluency on the day of PEF treatment (24 h after plating). Hoechst‐33342 (Ho) (2.25 μM) (catalog # H3570, Invitrogen, Thermo Fisher Scientific, Waltham, MA) dye, labeling the nuclei of all cells, was used to assess monolayer confluency and integrity before pulsing (see Figure S1).

Cryopreserved human‐induced pluripotent stem cell‐derived cardiomyocytes (hiPSC‐CMs) (catalog # 01434 ‐ iCell Cardiomyocytes^2^, Fujifilm Cellular Dynamics, Inc, Madison, WI) were stored in liquid nitrogen. Seven days prior to experimenters, cells were thawed according to the manufacturer's instructions and plated onto Matrigel‐coated 96‐well Nanofiber plates at a concentration of 115,000 cells per well to reach 100% cell confluency (see Figure S1). HiPSC‐CM cultures were maintained according to the manufacturer's recommendations at 37°C, with 5% CO_2_.

### 2.2 Experimental sample preparation

30 min before first pulse delivery, a 100% media change from the manufacturer's maintenance medium to a pulsing solution was performed. The pulsing solution (modified Tyrode solution) contained: 140 mM NaCl, 5 mM KCl, 2 mM CaCl_2,_ 2 mM MgCl_2_, 5 mM HEPES, 10 mM Glucose, and adjusted to a pH 7.4 using NaOH (Figure S1). 30 min after pulse delivery, a second 100% media change was performed with a maintenance medium to transfer the PEF‐treated monolayers into a cell incubator (37°C, with 5% CO_2_) until imaging. For hiPSC‐CMs, a serum‐free maintenance medium was used (catalog # M1038, Fujifilm Cellular Dynamics, Inc). To stain the irreversibly electroporated cells, a 100% media change was performed 30 minutes prior to imaging to add PI (15 μM) (Cat. #P3566, Invitrogen, Thermo Fisher Scientific), a small fluorescent molecule able to pass through cellular membrane pores >1.5 nm and bind to cells genetic material (Bowman et al., 2010). All experimental sample preparation and treatment timelines are reported in Figure S2. Prior to all media changes the replacement media was brought to 37°C.

### 2.3 Pulse delivery

**FIGURE 1:**
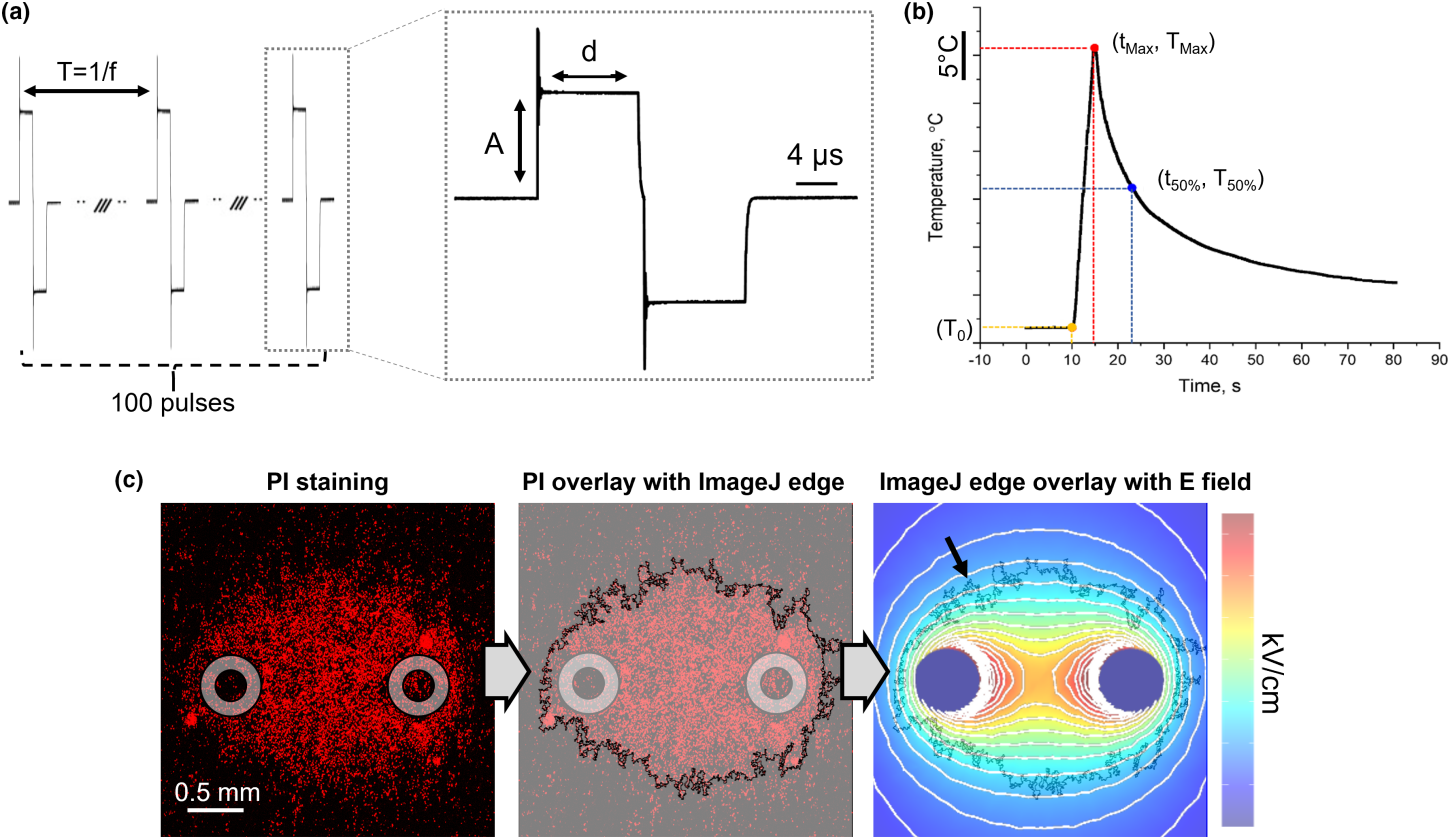
Definition of PEF parameters and experimental endpoints. (a) A representative PEF treatment (not in scale) composed of 100 biphasic pulses (d = 10 μs, 100 ns interphase delay, f = 1 kHz, A = 374 V). (b) A representative experimental temperature measure during a PEF treatment reporting the studied temperature endpoints. (c) An example of fluorescent staining by PI of an hESMC monolayer 4 h after PEF treatment (gray circles indicate the footprints of the electrodes); fluorescent images were analyzed using ImageJ to identify the outer edge of the IRE region and calculate its area; the IRE area was then compared with the electric field distribution generated by the electrodes positioned orthogonally to the cell monolayer for the determination of the EFT for cell death. See text for more details

An Anet A8 3D printer (Shenzhen Anet Technology Co) was modified to serve as an automated arm for accurate placement of a pair of stainless‐steel needle electrodes (0.61 mm diameter, 1.7 mm distance center to center), to facilitate the high‐throughput characterization of the PEF‐induced IRE areas in hESMC and hiPSC‐CM cultures (Casciola et al., 2020; Gudvangen et al., 2022). The heated stage of the 3D printer was used to maintain all wells of the 96‐well plate at 37 ± 1°C. A 2 kV custom FID GmbH voltage generator (model FPG 1B50‐1UL10, FID GmbH), controlled with a Berkeley Nucleonics digital delay generator (model 577‐4C, Berkeley Nucleonics Corporation), was used to apply the desired PEF treatment across the electrodes. A Tektronix oscilloscope (Tektronix) and a voltage probe (model P2501, Owon Technology Inc.) were used to measure the amplitude and duration of each PEF waveform applied to the sample. A schematic of the experimental setup is shown in Figure S3. For all experiments, a continuous sequence of 100 symmetric, biphasic, rectangular pulse signals with a 100 ns interphase delay was applied (Figure 1a). Treatment parameters were varied between samples including phase amplitude (A = 91, 190, 284, 372, 568 V), phase duration (d = 1, 5, 10 μs), and pulse repetition rate (f = 10, 100, 1000 Hz) inverse of the pulse repetition period (T). All possible combinations of treatment parameters were applied to hESMCs (*n* = 3), whereas for hiPSC‐CMs the phase amplitude was limited to 284 V (*n* = 3).

### 2.4 Measurements of temperature

A nonmetallic fiber optic STB probe (model L‐00‐14500‐01, Advanced Energy Industries) was used to continuously measure temperature in a cell‐free sample mimicking our in vitro models. To eliminate the influence of the STB probe on the PEF treatments, experiments used for monitoring temperature were separated from those used for performing the IRE analysis, as previously done (Arena et al., 2012). The STB probe response time is 0.25 s, the sampling rate is 0.02 s, and the diameter is 0.5 mm. As shown in Figure S3, the STB probe was positioned adjacent and parallel to one of the electrodes in order to capture the temperature increase in the proximity of the hottest zone as a function of time. Prior to pulsing, 100 μl of Tyrode was allowed to stabilize to an initial temperature, (*T*_0_) 37 ± 1°C. Once a stable temperature was reached, the measurement was initiated, and the pulse was delivered ~10 s after. Temperature measurements were recorded continuously for 1–5 min to allow the media to recover to at least 50% of the maximum change in temperature. Calculations of the maximum temperature change (∆T_Max_) and 50% recovery time (t_50%_) were made according to equations 1 and 2. A representative temperature–time curve is presented in Figure 1b.

(1) $$∆T_{\mathrm{Max}}=T_{\mathrm{Max}}-T_{0},$$

(2) $$t_{50\%\text{recovery}}=t_{\mathrm{Max}}-t_{50\%}.$$

### 2.5 Fluorescent imaging

Fluorescent images of the IRE regions were captured with a laser scanning confocal microscope (FluoView 3000, Olympus America) using a 4× dry objective with a numerical aperture of 0.16. PI emission was excited with a 561 nm laser and detected in the wavelength range of 570–670 nm. All camera and laser settings were kept constant across experiments.

### 2.6 Image analysis and area calculation

All fluorescent images were analyzed with ImageJ software (NIH) (Schneider et al., 2012) according to the following methodology. First, the contrast was adjusted twice, allowing 0.3% of the pixel to be saturated. Then, images were converted to an 8‐bit binary format (background threshold 14 ± 2%). Stacks of binary images were used as input to the Analyze Particle function that identified and quantified the area of the PI‐stained regions (Figure 1c). Holes in the outlined region were automatically filled by the Analyze Particle function. To exclude dead cells embedded in the monolayer outside the IRE region, the minimum particle size was set to be 2000 pixel^2^ (i.e., ~0.08 mm^2^). After the IRE area was quantified, the electrodes' imprint area was subtracted from the IRE area when needed.

### 2.7 Theoretical IRE EFT calculation

A computational model was used to determine the theoretical relationship between the size of the IRE region and the electric field distribution at the cell monolayer in order to estimate the IRE EFT (Figure 1c), as previously described (Arena et al., 2012; Aycock et al., 2021; Aycock et al., 2022; Liu et al., 2021; Neal et al., 2014). The pulsing medium and electrodes 3D geometry were constructed in the finite element analysis software COMSOL Multiphysics 5.6 (COMSOL Inc., Stockholm, Sweden) to model the electric field distribution under static conditions using a fine mesh consisting of 166,212 elements (Figure S3). The pulsing medium was assigned an electrical conductivity of 2.3 S/m (i.e., the value measured at 37°C). An electric potential equal to the phase amplitudes of the PEF treatments was applied to one electrode, whereas the other was set to 0 V. Electric field contours at varying magnitudes were created, and the surface area contained within each contour was integrated with 1 V/cm steps. Similar to (Aycock et al., 2022), the curve‐fitting tool in MATLAB (MathWorks Inc.) was used to fit a two‐term exponential equation to the resulting area versus electric field data. Finally, measured IRE areas were used as inputs to this equation to compute EFTs.

## 3 RESULTS

### 3.1 HiPSC‐CMs display larger IRE regions relative to HESMCs

**FIGURE 2:**
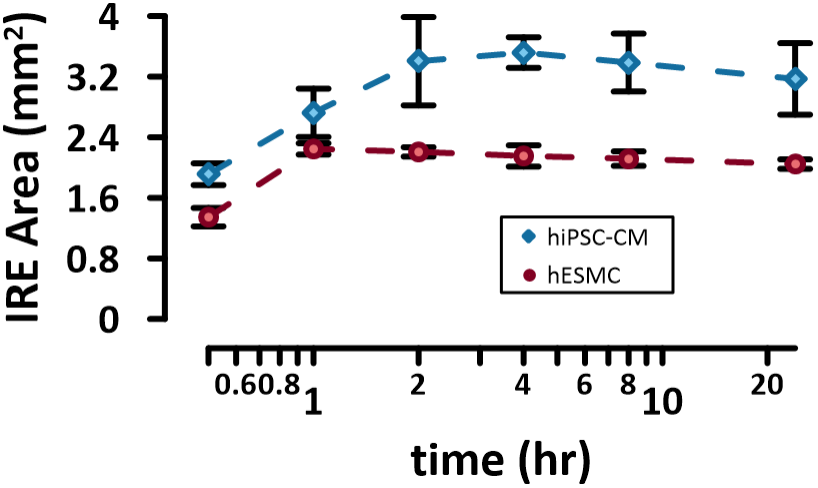
IRE area measured at different time intervals after PEF treatment to determine the timepoint for cell death in hiPSC‐CMs and hESMCs. HiPSC‐CMs and hESMCs were exposed to a train of 100 pulses, d = 5 μs, f = 10 Hz, and A = 236 and 372 V, respectively. Monolayers were stained with PI at different time points after PEF treatment to assess the IRE region over time. See text for more details. The error bars represent the standard error for a sample size of *n* = 3–4.

Various time points were evaluated to determine the most consistent assay window to investigate irreversible cell electroporation (i.e., cell death). PI‐stained images were acquired 0.5, 1, 2, 4, 8, and 24 h after PEF treatments in both hESMCs and hiPSC‐CMs. The IRE region displayed an increase in PI area uptake from 0.5 to 1–4 h (Figure 2). At timepoints longer than 4 h, the IRE area stabilized or decreased as a consequence of proliferating hESMCs or lifting of the detachment of dead cells in both cell types. As such, the work described here will focus on the IRE effects 4 h after treatment.

**FIGURE 3:**
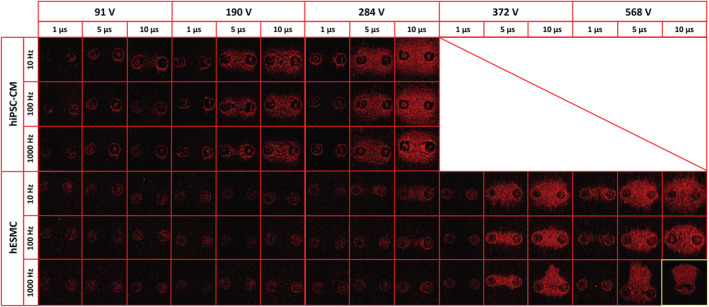
Fluorescent staining by PI to assess IRE regions in hiPSC‐CMs and hESMCs following PEF treatments. Representative images showing IRE regions in hiPSC‐CMs and hESMCs identified by PI staining are reported for all sets of PEF parameters tested in this study. As the phase duration, phase amplitude, and pulse repetition period increased, the IRE region surrounding the electrode imprint increased. For all combinations of pulse parameters, hiPSC‐CMs showed PI uptake at lower PEF doses than hESMCs. One image (d = 10 μs, f = 1000 Hz, A = 568 V) is highlighted in yellow to show that it is on a scale 4x the size of the other images. See text for more details

Our results show the dependency of the IRE areas on the PEF parameter combinations applied to hiPSC‐CMs and hESMCs (Figure 3). While hESMCs were exposed to all PEF combinations including A = 91, 190, 284, 372, and 568 V, hiPSC‐CMs were exposed to treatments up to A = 284 V since at higher doses hiPSC‐CMs dissociated and monolayers were damaged. For hESMCs, asymmetric regions were observed at high PEF doses including (A = 372 V, f = 1000 Hz, d = 10 μs), (A = 568 V, f = 1000 Hz, d = 5 μs), and (A = 568 V, f = 1000 Hz, d = 10 μs). Additional experiments (data not shown) indicate that this asymmetry, emphasized by the high phase voltage applied, could be ascribed to the minor inclination of the electrodes with respect to the plane of the cell monolayer. These regions were not used for analysis.

**FIGURE 4:**
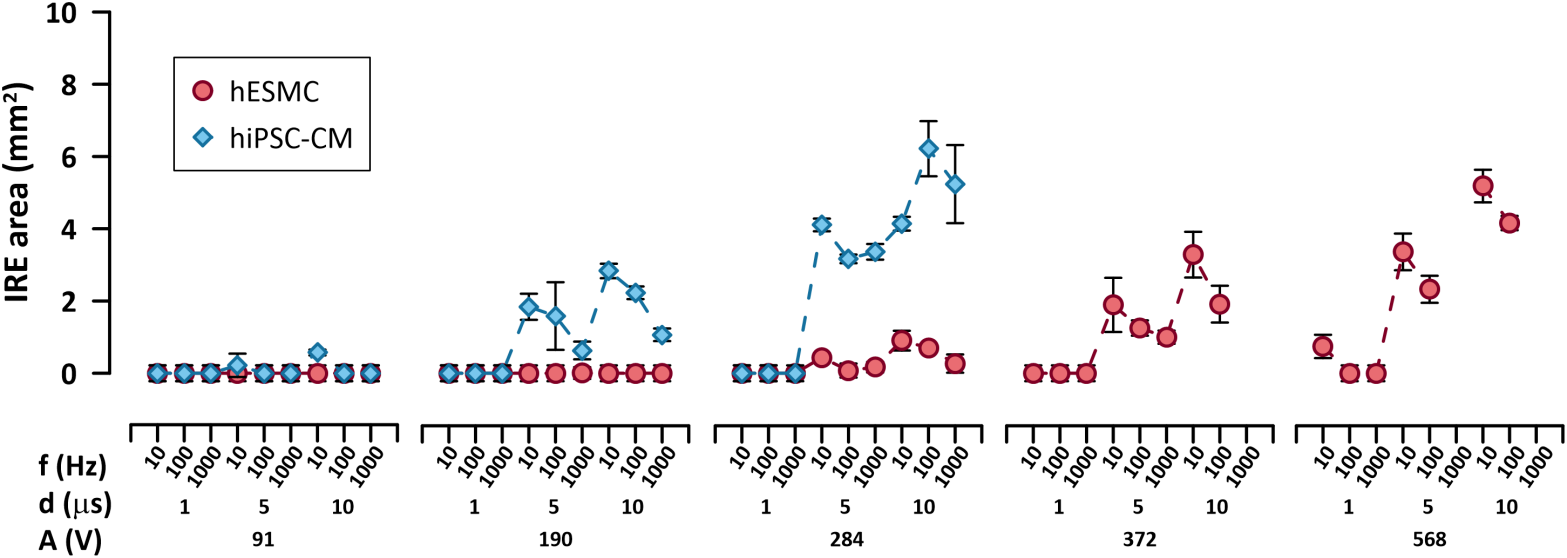
IRE areas by pulsed electric field treatments with varying pulse parameters in hiPSC‐CMs and hESMCs. Area measurements of the IRE regions 4 h after treatment for both hiPSC‐CMs and hESMCs for all combinations of treatment parameters are presented. Trains of 100 pulses with A = 91, 190, 284, 372, 568 V (left to right panels) and d = 1, 5, 10 μs were applied at f = 10, 100, 1000 Hz. The highest phase amplitude applied to hiPSC‐CMs was 284 V. IRE areas were quantified from the borders of PI uptake 4 h after PEF treatment. See text for more details. The error bars represent the standard error for a sample size of *n* = 3 for all data points

PEF treatment, for the symmetric IRE regions, resulted in a positive correlation between the area of the IRE region and increasing amplitude and duration (Figure 4). Conversely, increasing pulse reptation rates resulted in reduced IRE areas. Treatments that did not produce any irreversible electroporation with an area corresponding to the size of the imprint of the electrodes (i.e., 1.05 ± 0.04 mm^2^) were reported as 0 mm^2^. When comparing the IRE regions of hESMCs and hiPSC‐CMs, the latter displayed significantly larger IRE areas for identical treatments compared with hESMCs (Figure 4). For the set of PEF parameters investigated, the PI uptake beyond the electrode imprint was initially observed in hiPSC‐CMs for the treatment of A = 91 V, f = 10 Hz, and d = 5 μs while region formation for hESMCs was not observed until the phase amplitude was increased threefold (i.e., A = 284 V) for the same treatment. These results demonstrate that hiPSC‐CMs show larger IRE areas than hESMCs for the same pulsing conditions.

### 3.2 HESMCs display higher EFTs compared with HiPSC‐CMs

We next used the IRE region to quantify the IRE EFT in both hESMCs and hiPSC‐CMs. Significantly lower electric field exposure was required to induce cell death in hiPSC‐CMs compared with hESMCs (Table 1). Cell death for hiPSC‐CMs was observed at EFTs as low as 0.65 ± 0.01 kV/cm at a treatment of d = 10 μs, and f = 10 Hz, and A = 284 V. The electric field required to induce cell death in hESMCs for the same treatment was determined to be 1.67 ± 0.12 kV/cm. The ratio hESMC/hiPSC‐CM IRE EFT was approximately 2–2.5 folds for the treatment tested. These results demonstrate that hiPSC‐CMs have lower EFTs than hESMCs.

**TABLE 1:** Summary table reporting endpoints for PEF treatments with varying pulse parameters in hiPSC‐CMs and hESMCs

A (V)	d (μs)	f (Hz)	ΔT_Max_ (°C)	t_50%_ (s)	IRE area hiPSC‐CM (mm^2^)	EFT hiPSC‐CM (kV/cm)	IRE area hESMC (mm^2^)	EFT hESMC (kV/cm)
91	1	10	<1		n/a	n/a	n/a	n/a
91	1	100	<1		n/a	n/a	n/a	n/a
91	1	1000	<1		n/a	n/a	n/a	n/a
91	5	10	1.0 ± 0.1	7.2 ± 0.8	0.02 ± 0.17	n/a	n/a	n/a
91	5	100	1.6 ± 0.1	4.0 ± 0.4	n/a	n/a	n/a	n/a
91	5	1000	1.7 ± 0.1	3.2 ± 0.3	n/a	n/a	n/a	n/a
91	10	10	2.1 ± 0.1	6.7 ± 0.3	0.69 ± 0.50	0.76 ± 0.16	n/a	n/a
91	10	100	2.1 ± 0.1	3.5 ± 0.3	0.14 ± 0.29	n/a	n/a	n/a
91	10	1000	2.3 ± 0.1	2.8 ± 0.1	n/a	n/a	n/a	n/a
190	1	10	< 1		n/a	n/a	n/a	n/a
190	1	100	1.0 ± 0.1	3.1 ± 0.3	n/a	n/a	n/a	n/a
190	1	1000	1.1 ± 0.1	3.2 ± 0.3	n/a	n/a	n/a	n/a
190	5	10	3.1 ± 0.1	6.0 ± 0.3	2.4 ± 0.030	0.80 ± 0.01	n/a	n/a
190	5	100	4.7 ± 0.1	2.9 ± 0.2	1.93 ± 0.52	0.93 ± 0.16	n/a	n/a
190	5	1000	5.9 ± 1.8	2.4 ± 0.2	1.44 ± 0.34	1.10 ± 0.14	n/a	n/a
190	10	10	5.9 ± 0.1	6.0 ± 0.2	2.86 ± 0.12	0.71 ± 0.02	n/a	n/a
190	10	100	8.4 ± 0.2	2.5 ± 0.2	2.43 ± 0.52	0.80 ± 0.13	n/a	n/a
190	10	1000	7.9 ± 0.1	2.7 ± 0.1	1.92 ± 0.60	0.94 ± 0.18	n/a	n/a
284	1	10	2.8 ± 0.1	6.8 ± 0.2	0.16 ± 0.27	n/a	n/a	n/a
284	1	100	4.1 ± 0.1	3.5 ± 0.1	0.36 ± 0.13	n/a	n/a	n/a
284	1	1000	4.4 ± 0.2	3.2 ± 0.1	n/a	n/a	n/a	n/a
284	5	10	6.6 ± 0.2	5.4 ± 0.1	3.71 ± 0.65	0.90 ± 0.14	0.27 ± 0.13	n/a
284	5	100	9.3 ± 0.2	2.0 ± 0.2	3.38 ± 0.23	0.96 ± 0.06	0.01 ± 0.02	n/a
284	5	1000	8.2 ± 0.5	2.3 ± 0.1	3.14 ± 0.18	1.02 ± 0.05	n/a	n/a
284	10	10	12.5 ± 0.2	5.5 ± 0.4	6.50 ± 0.17	0.65 ± 0.02	1.41 ± 0.22	1.67 ± 0.12
284	10	100	19.7 ± 0.8	2.4 ± 0.5	6.11 ± 0.76	0.71 ± 0.06	0.55 ± 0.12	n/a
284	10	1000	11.7 ± 0.6	5.9 ± 0.4	5.12 ± 1.09	0.77 ± 0.12	0.19 ± 0.13	n/a
372	1	10	4.6 ± 0.1	6.4 ± 0.3	n/a	n/a	0.53 ± 0.17	n/a
372	1	100	6.7 ± 0.2	3.1 ± 0.1	n/a	n/a	0.01 ± 0.01	n/a
372	1	1000	6.8 ± 0.1	2.8 ± 0.2	n/a	n/a	0.01 ± 0.03	n/a
372	5	10	14.4 ± 0.3	5.7 ± 0.2	n/a	n/a	2.18 ± 0.25	1.72 ± 0.12
372	5	100	17.2 ± 0.7	2.4 ± 0.2	n/a	n/a	1.34 ± 0.12	2.17 ± 0.08
372	5	1000	9.5 ± 0.4	4.2 ± 0.5	n/a	n/a	1.01 ± 0.12	2.39 ± 0.08
372	10	10	24.0 ± 1.3	6.1 ± 1.1	n/a	n/a	3.50 ± 0.27	1.24 ± 0.08
372	10	100	30.5 ± 1.2	8.6 ± 0.5	n/a	n/a	2.13 ± 0.24	1.74 ± 0.12
372	10	1000	20.3 ± 2.8	13.3 ± 1.2	n/a	n/a	Asymmetric	n/a
568	1	10	7.8 ± 0.4	6.4 ± 0.4	n/a	n/a	1.14 ± 0.13	3.16 ± 0.10
568	1	100	10.4 ± 0.4	2.6 ± 0.2	n/a	n/a	0.19 ± 0.20	n/a
568	1	1000	9.7 ± 0.2	3.4 ± 0.3	n/a	n/a	0.05 ± 0.10	n/a
568	5	10	21.5 ± 0.9	5.4 ± 0.1	n/a	n/a	3.55 ± 0.12	1.85 ± 0.05
568	5	100	21.7 ± 0.6	2.9 ± 0.6	n/a	n/a	2.35 ± 0.20	2.38 ± 0.11
568	5	1000	19.0 ± 2.2	14.9 ± 0.6	n/a	n/a	Asymmetric	n/a
568	10	10	29.6 ± 2.9	15.6 ± 1.4	n/a	n/a	5.11 ± 0.44	1.39 ± 0.10
568	10	100	30.3 ± 1.8	15.5 ± 1.2	n/a	n/a	4.07 ± 0.27	1.67 ± 0.09
568	10	1000	n/a	n/a	n/a	n/a	Asymmetric	n/a

*Note:* For all combinations of pulsed electric field parameters tested, we report the measured maximum temperature change (ΔT_Max_), the 50% recovery time (t_50%_), the IRE area, and the IRE EFT for hiPSC‐CMs and hESMCs. The IRE area was calculated by subtracting the area of the electrode imprint. Areas equal to the electrode imprint are not reported (n/a). IRE EFTs were calculated only for IRE areas larger than 0.60 mm^2^. For all the endpoints tabled, we report the average and standard error (95% confidence interval) of *n* = 3 independent measures.

### 3.3 PEF modulates temperature

In order to assess the potential thermal gradients during PEF treatments, we measured the maximum temperature change, and 50% recovery time for all the tested parameters (Table 1). In most of our experiments, the temperature increased between 2 and 13°C, reaching values up to 30°C at the highest PEF doses. Our data indicated a positive correlation between increasing temperature and increasing phase amplitude and duration. The temperature change observed with respect to pulse repetition rate showed a parabolic trend with the highest value observed at a pulse repetition rate of 100 Hz. This suggests that the temperature probe may underestimate the highest temperature increase for high repetition rates (e.g., f = 1000 Hz). These results confirm that temperature changes in vitro are dependent on the specific combination of selected PEF parameters.

## 4 DISCUSSION

Here, for the first time, we report an in vitro model that uses a 2D cell monolayer format to characterize PEF response in two different human cell types, hESMCs and hiPSM‐CMs, across a range of clinically relevant treatment parameters. The characterization highlights the cell‐specific nature of PEF‐based treatments in human cardiac and esophageal models by showing that, for the same PEF treatment, IRE EFTs for hiPSC‐CMs are significantly lower compared with that of hESMCs (i.e., 2–2.5‐fold factor). Additionally, our results demonstrate that temperature changes were dependent on the specific combination of treatment parameters. Temperature changes ranged from less than 1°C to as high as 30°C. Nevertheless, all temperature changes were transient, and the highest observed increase returned to 50% of the maximum in less than tens of seconds.

### 4.1 Cell‐specific PEF treatments demonstrated for HESMCs and HiPSC‐CMs

Selective treatments, particularly with respect to the ability to induce cell death in hiPSC‐CMs while leaving hESMCs largely unaffected have immediate clinical relevance for the treatment of arrhythmias (Howard et al., 2020). The current clinical standard for treating drug‐resistant arrhythmia comes with the rare but significant adverse effects of off‐target tissue damage, particularly in the adjacent esophagus, which is often fatal (Calkins et al., 2009). The results show that hiPSC‐CMs are significantly more sensitive to PEF treatments compared with hESMCs illustrating that PEFs could be a tissue‐specific treatment alternative to current ablation modalities and may present less off‐target tissue damage risk. More specifically, our results demonstrate that to produce an IRE region with a comparable area in both cell types, 2 times higher phase amplitude must be applied to hESMCs than hiPSC‐CMs, when the same PEF treatments were used. For example, for d = 5 μs and f = 10 Hz treatment, the IRE area in hESMCs was 2.18 ± 0.25 mm^2^ at 372 V and in hiPSC‐CMs was 2.4 ± 0.03 mm^2^ at 190 V. When considering cell death thresholds, the lowest IRE EFT was 0.65 ± 0.01 kV/cm for hiPSC‐CMs and 1.24 ± 0.07 kV/cm for hESMCs at the treatment of d = 10 μs, f = 10 Hz. Generally, for the PEF treatments tested, IRE EFTs for hESMCs were approximately 2 and 2.5 higher than for hiPSC‐CMs. Cell morphology varies between cell types and plays a major role in the transmembrane potential variations induced by PEF and contributing to cell death EFT values, mostly for longer pulse durations (Agarwal et al., 2007; Henslee et al., 2011). HiPSC‐CMs were slightly larger than hESMCs (see Figure S1) possibly contributing to lower IRE EFTs. However, other factors, such as electric properties (Schoenbach et al., 2001) and nucleus‐to‐cellular area ratio (Aycock et al., 2022), have been shown to impact cell selectivity, especially for waveforms with extremely short pulse durations. Additional studies will be needed to elucidate the mechanisms for PEF selectivity in cardiac and esophageal cells.

In addition to the relevance for the treatment of arrhythmia with PEFs, the in vitro tool presented here supports a broader impact, on the development of PEF‐based technologies by device developers. In line with other studies, in this work, we observed that specific combinations of treatment parameters will lead to vastly different IRE region sizes and EFTs dependent on the selected parameters and cell type. When developing PEF‐based devices developers will need to consider the intended application in order to optimize the selection of treatment parameters for its intended purpose. The characterization here provides initial results on how specific pulse parameters affect the size of the IRE region and the EFT for two different clinically relevant cell types. The development of PEF‐based devices for the treatment of drug‐resistant arrhythmia might consider similar characterizations to optimize the selection of PEF parameters to produce a specific IRE region size at an EFT that selectively kills hiPSC‐CMs while leaving hESMCs unaffected and minimizing temperature changes.

### 4.2 Temperature changes strongly dependent on treatment parameter combination

The 2D model proposed here may also be useful for the qualitative prediction of the thermal characteristics for a specific combination of treatment parameters. Likewise, near the electrodes, where the highest electric field amplitude is reached, we observed significant temperature changes for higher PEF doses. PEF‐induced temperature changes ranged from less than 1°C to as high as 30°C, however, the highest observed ∆T returned to 50% of the maximum recorded temperature in less than tens of seconds. While the quantitative assessment of the actual thermal damage on cells exposed to PEF was outside the scope of this paper, our results suggest a wide range of temperatures observed for different combinations of treatment parameters highlighting the importance of evaluating temperature data for treatments where changes in temperature are a concern. A greater understanding of how specific treatment parameter combinations affect temperature for PEF‐based treatments may become an essential aspect of the device and treatment development process.

### 4.3 Current limitations and future impacts

Perhaps one of the biggest advantages for the development of PEF‐based devices, and the most difficult hurdles in their regulation, is the wide range of pulse parameters that can be modulated for the intended PEF application. Significant research to characterize the effects of all combinations of PEF parameters could aid the development process. Here, only changes in amplitude, duration, and frequency were considered while holding all other PEF parameters constant. Additional parameter combinations that likely have effects on the resulting EFTs and temperature characteristics include biphasic vs monophasic, symmetrical vs asymmetrical waveforms, number of pulses, interphase delay, and number of pulse packages/trains, among others. More extensive studies that evaluate all possible combinations of pulse parameters will be required to better understand the production of novel PEF‐based devices. Moreover, this study did not consider pulse repetition rates over 1000 Hz or durations under 1 μs which should be considered for evaluating the production of HFIRE and nanosecond PEF devices.

Second, the results here are for a 2D in vitro model while nearly all devices are being developed for applications involving 3D tissues. Although the results presented do not determine the exact electrophysiological and thermal relationship between PEF‐based IRE in 2D and 3D, a correlation between 2D and 3D is expected. With proper characterization and comparison between similar 2D and 3D models, a calibration curve may be created to predict the thermal characteristics, size of the expected IRE region, and corresponding EFT for a specific treatment in 3D using the results for an identical treatment in our 2D in vitro model. The ability to use a 2D model to predict the size of the ablation lesion in 3D tissue will be important for the development of novel PEF‐based devices. 2D in vitro models are expected to be faster, providing the ability to rapidly characterize different organ‐specific cell types. The high‐throughput characterization of the 2D model will allow for accelerated PEF treatment optimization and device development which will ultimately result in an improved patient experience. In addition, the 2D model will provide a simple, cost‐effective methodology, requiring less expensive equipment, and less technical training all while using readily available commercial reagents and cell lines when compared to other 3D tissue methodologies.

Another noteworthy study limitation was that no functional assessment (i.e., action potential propagation or calcium waves) of the cardiac monolayers was performed. As the primary goal was to contrast cardiac and esophageal cells' response to PEF, the “terminal” endpoint (irreversible electroporation detected through PI staining) was selected for the analysis. Cardiac monolayers might be “functionally ablated” (i.e., stop transmitting action potentials, etc.) at different, likely lower, PEF exposures. Furthermore, PEF‐induced electrical stimulation and reversible electroporation were also not considered, even though they can be achieved at lower EFTs than cell death (Gudvangen et al., 2022). Future studies are needed to detect PEF effects that were omitted in this tissue‐specific focused report.

Finally, here we used standard commercially available 2D hiPSC‐CM cultures with a mixed population of hiPSC‐CMs from each cardiac subtype (i.e., ventricular, atrial, and nodal) (Ma et al., 2011). Future studies may benefit from the application of chamber‐specific models, for example, to assess PEF ablation of atrial or ventricular myocytes.

## 5 CONCLUSIONS

Our results provide the first IRE characterization of hiPSC‐CMs and hESMCs under parallel experimental conditions, using a novel 2D in vitro approach. The characterization highlights the cell‐specific nature of PEF‐based treatments in human cardiac and esophageal models by showing that for the set of PEF parameters investigated, IRE EFT for hESMCs was approximately between 2 and 2.5 folds higher than hiPSC‐CMs.

## DISCLAIMER

This article reflects the views of the authors and should not be construed to represent the US Food and Drug Administration's views or policies. The mention of commercial products, their sources, or their use in connection with material reported herein is not to be construed as either an actual or implied endorsement of such products by the Department of Health and Human Services.

## AUTHOR CONTRIBUTIONS

M.C., D.K., T.F., and K.B. conceived and designed the research. D.K. and M.C. performed experiments and analyzed data. M.C. performed electric field modeling. M.C., D.K., and K.B interpreted the results of the experiments. M.C., D.K., and K.B. drafted the manuscript. T.K. edited and revised the manuscript. M.C., D.K., T.F., and K.B. approved the final version of the manuscript.

## FUNDING INFORMATION

The study was supported by the U.S. Food and Drug Administration, Office of Science and Engineering Laboratories, and the Center for Devices and Radiological Health Critical Path grants (to M.C., T.F., and K.B.).

## CONFLICT OF INTEREST

The authors declared no competing interest in this work.

## Supporting information

Online Supplement
