# Supplementary material for: Human cardiomyocytes are more susceptible to irreversible electroporation by pulsed electric field than human esophageal cells
Source: Physiol Rep. 2022 Oct 27;10(20):e15493. doi: 10.14814/phy2.15493 (PMC9612150; doi:10.14814/phy2.15493)
Supplement: Supplementary file 1 — Online Supplement [file PHY2-10-e15493-s001.docx]

***Cell Culture and Maintenance***

Hoechst-33342 (Ho) dye, labeling the nuclei of all cells, was used to assess monolayer confluency and integrity before pulsed electric field (PEF) treatments (Supplementary Figure 1) in human induced pluripotent stem cell cardiomyocytes derived (hiPSC-CM) and human esophageal smooth muscle cells (hESMC).

HiPSC-CMs were plated at a concentration of 115,000 cells per well following manufacturer’s instructions and adjusting the concentration to account for increased surface in 96-well Nanofiber plates. To assess confluency of hESCM monolayers over time, cells were plated on plastic bottom 96-well plates at different concentrations per well. Bright field images were acquired and analyzed every 2 hr with IncuCyte (Sartorius, Germany). For 75k cells per well, 100% confluency was reached 4 hr after plating and maintained for the duration of the experiment (i.e., 24 hr). In this study, we used 100,000 cells per well to account for increased surface in 96-well Nanofiber plates.


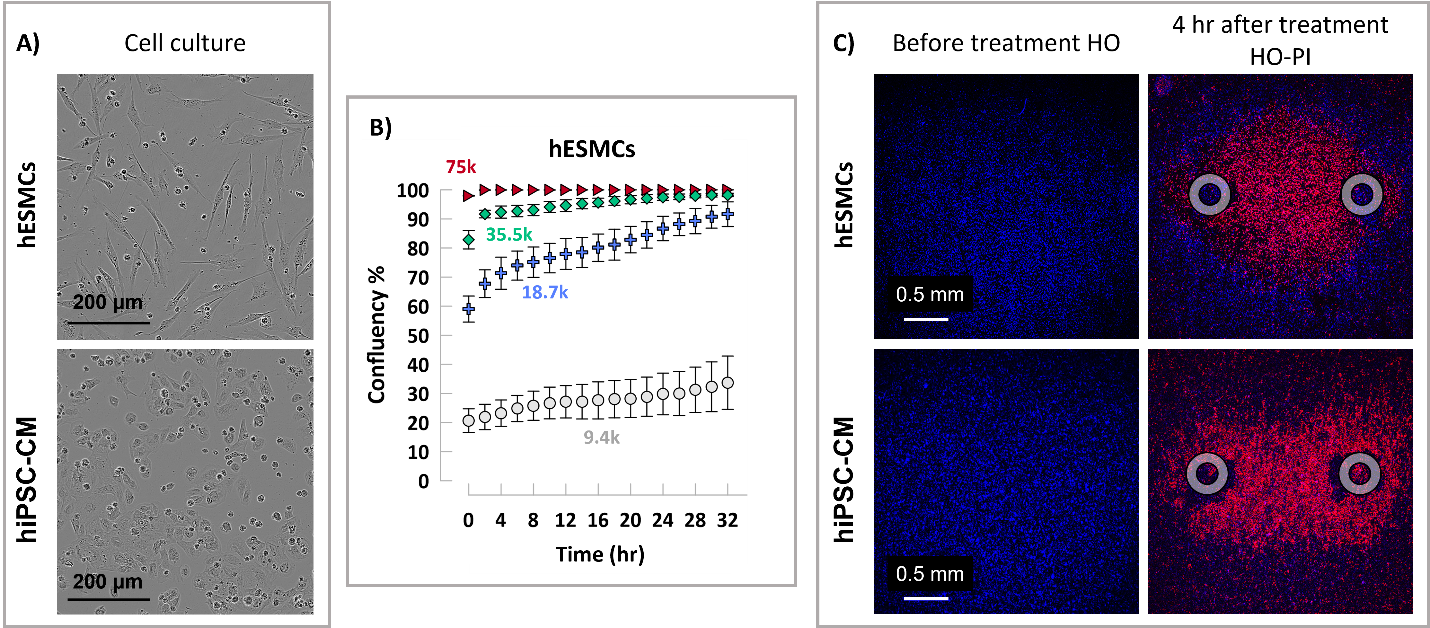


**Supplementary Figure 1: Monolayer confluency and integrity assessment prior PEF treatment**

A) Representative bright field images of hESMC and hiPSC-CM cultures obtained with IncuCyte. B) Shows hESMCs confluency % over time for different numbers of cells per well at plating (i.e., 0 hr). N = 3-6. C) Representative, fluorescence images obtained with confocal microscopy show 100% confluency and integrity of the hESMC and hiPSC-CM monolayers before treatment: (Left) Cell nuclei were stained with Ho (blue) and imaged before PEF treatment; (right) irreversibly electroporated cells were stained with Propidium Iodide (PI, red) and imaged 4 hr after treatment.

***Sample preparation and irreversible electroporation (IRE) treatment delivery – timeline***


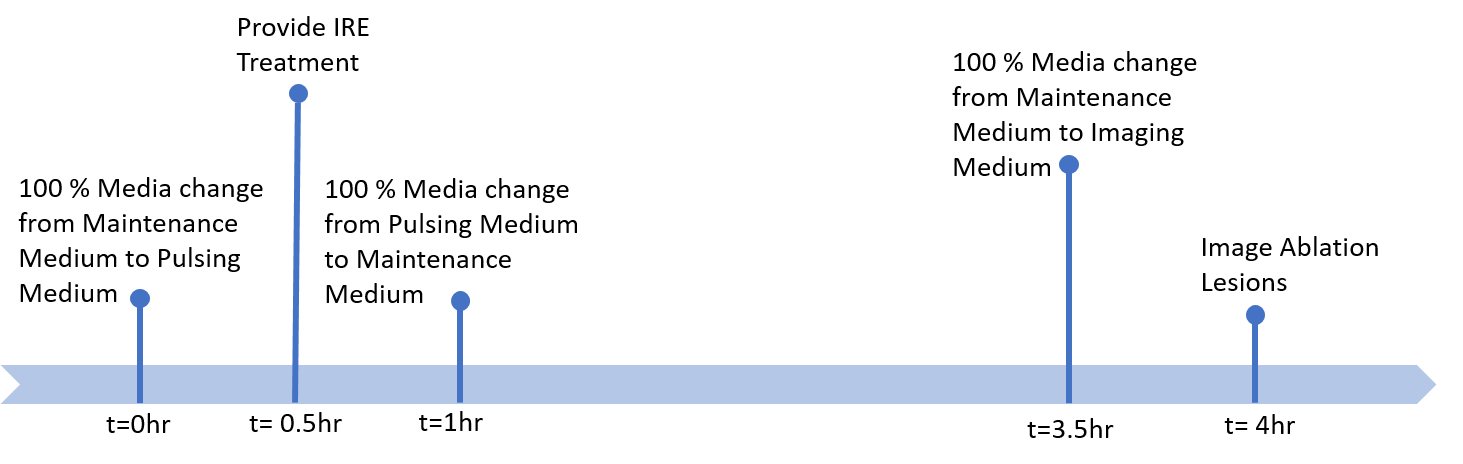


**Supplementary Figure 2: Experimental sample preparation for PEF treatment and analysis**

Timeline of all media changes performed throughout the experiment. Cells were cultured in maintenance medium that was replaced by pulsing medium (modified Tyrode solution) 0.5 hr before PEF treatments were delivered. 30 minutes after pulse delivery a second 100% media change was performed to transfer the PEF treated monolayers in maintenance medium into a cell incubator until imaging. To image dead cells due to PEF treatments, a 100% media change was performed 30 minutes prior imaging to stain irreversibly electroporated cells using imaging medium composed by modified Tyrode solution and PI.

***Experimental set up for high-throughput PEF delivery to cell monolayers***
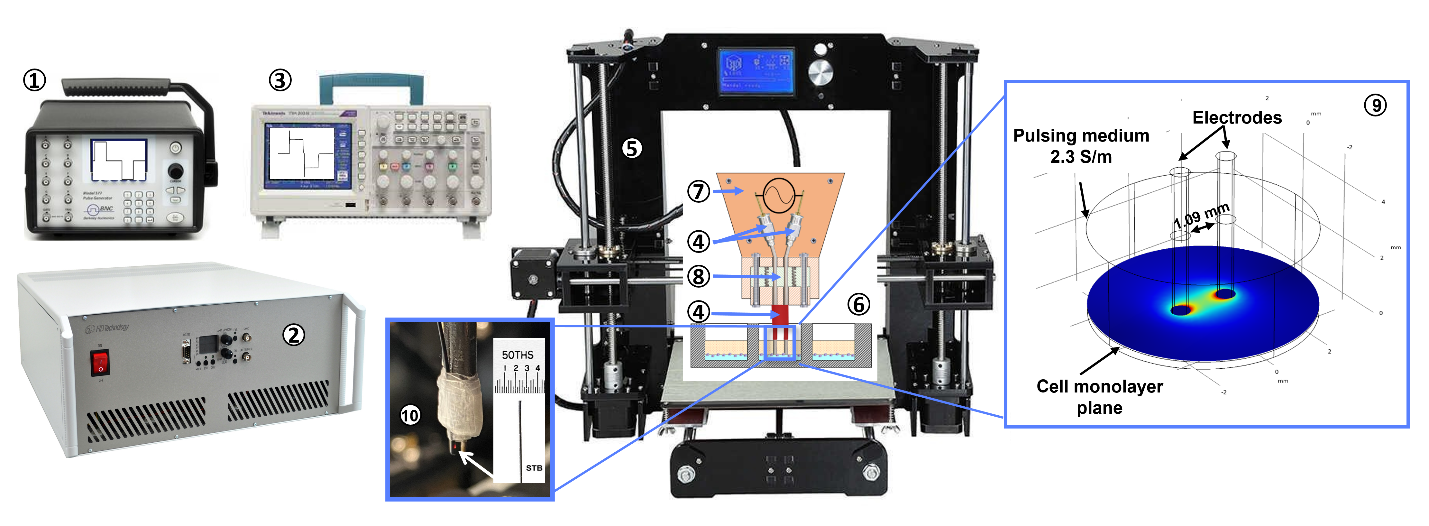


**Supplementary Figure 3: Schematic of the experimental setup**

A digital delay generator (1) was used to trigger a custom PEF voltage generator (2). An oscilloscope (3) with attenuation 1:100 was used to measure the waveform applied at the load between the needle electrodes (4). A 3D printer (5) was utilized as a robotic arm for accurate placement of the electrodes onto the multi well plate (6). The electrodes were supported by a holder (7) equipped with a spring system (8) to minimize the contact of the electrodes with the bottom of the multi well plate. The electric field distribution produced by the needle electrodes orthogonal to the cell monolayer was computed with numerical simulations mimicking the experimental geometry and materials (9). The temperature increase due to PEF treatments was monitored using an optic probe (10) placed between the electrodes, adjacent to one of them.
